# Supplementary material for: Revisiting the Trans-Ancestry Genetic Correlation of Refractive Error
Source: Invest Ophthalmol Vis Sci. 2025 Aug 26;66(11):60. doi: 10.1167/iovs.66.11.60 (PMC12395803; doi:10.1167/iovs.66.11.60)

Revisiting the Trans-Ancestry Genetic Correlation of Refractive Error

Supplementary Information

Supplementary Note S1. Adjustment for sources of bias by the TAGC-UDR method when calculating trans-ancestry genetic correlation.

The SNP-heritability (h^2_{SNP}) in the EUR sample determines the variance in refractive error that would be explained or predicted by a polygenic score in a (large) EUR evaluation sample if the SNP effects $\hat{\beta}$ in the EUR population were estimated without error. However, because the sample size of the EUR GWAS sample (Figure 1A) is not infinitely large, the noisy SNP effects $\hat{\beta}$ will downwardly bias the variance in refractive error predicted by a polygenic score in a EUR evaluation sample. This source of bias is termed '#1' in the legend of Figure 1.

When the EUR-derived polygenic score is applied to a non-EUR evaluation sample, two additional sources of bias arise. The source of bias termed '#2' in the legend of Figure 1 is similar in nature to source #1; it arises when estimating the genetic effects $\hat{\alpha}$ in the non-EUR evaluation sample as a result of the small sample size of the non-EUR evaluation sample. The source of bias termed '#3' in the legend of Figure 1 also affects the accuracy of gauging the genetic effects $\hat{\alpha}$ in the non-EUR evaluation sample. However, this time the bias arises from differences in LD patterns between the EUR and non-EUR sample (Figure 1B) rather than due to the small sample size.

The TAGC-UDR method estimates the degree of downward bias introduced from sources #1, #2 and #3, then upscales the genetic correlation estimate $G\beta\alpha$ to account for all of these sources of bias.

It is also possible that the h^2_{SNP} of refractive error is different in the EUR sample compared to the non-EUR sample. For example, if there is a difference in the time children spend outdoors between the two populations, then the non-genetic contribution to the variance in refractive error in the two populations may differ, which will have a knock-on effect of changing the heritability. When running a TAGC-UDR analysis, the user inputs h^2_{SNP} values for the two populations; the later steps of the TAGC-UDR analysis assume these h^2_{SNP} values are accurate. The TAGC-UDR method accounts for a difference in h^2_{SNP} in the two populations, if present, by further up or down-scaling the estimate of $G\beta\alpha$. However, estimating h^2_{SNP} in the non-EUR population is challenging due to the small sample size of the non-EUR evaluation sample. In the current work, we addressed this issue by calculating trans-ancestry genetic correlation estimates across a range of plausible h^2_{SNP} values, to provide an indicative range for the true genetic correlation.

Figure S1. Trans-ancestry genetic correlation estimates. Error bars correspond to 95% confidence intervals. The EUR-EUR comparison was included as a calibration factor with the expectation that this would provide a genetic correlation $r_g \approx 1.0$. Abbreviations: AFR = African, EAS = East Asian, EUR = European, SAS = South Asian.

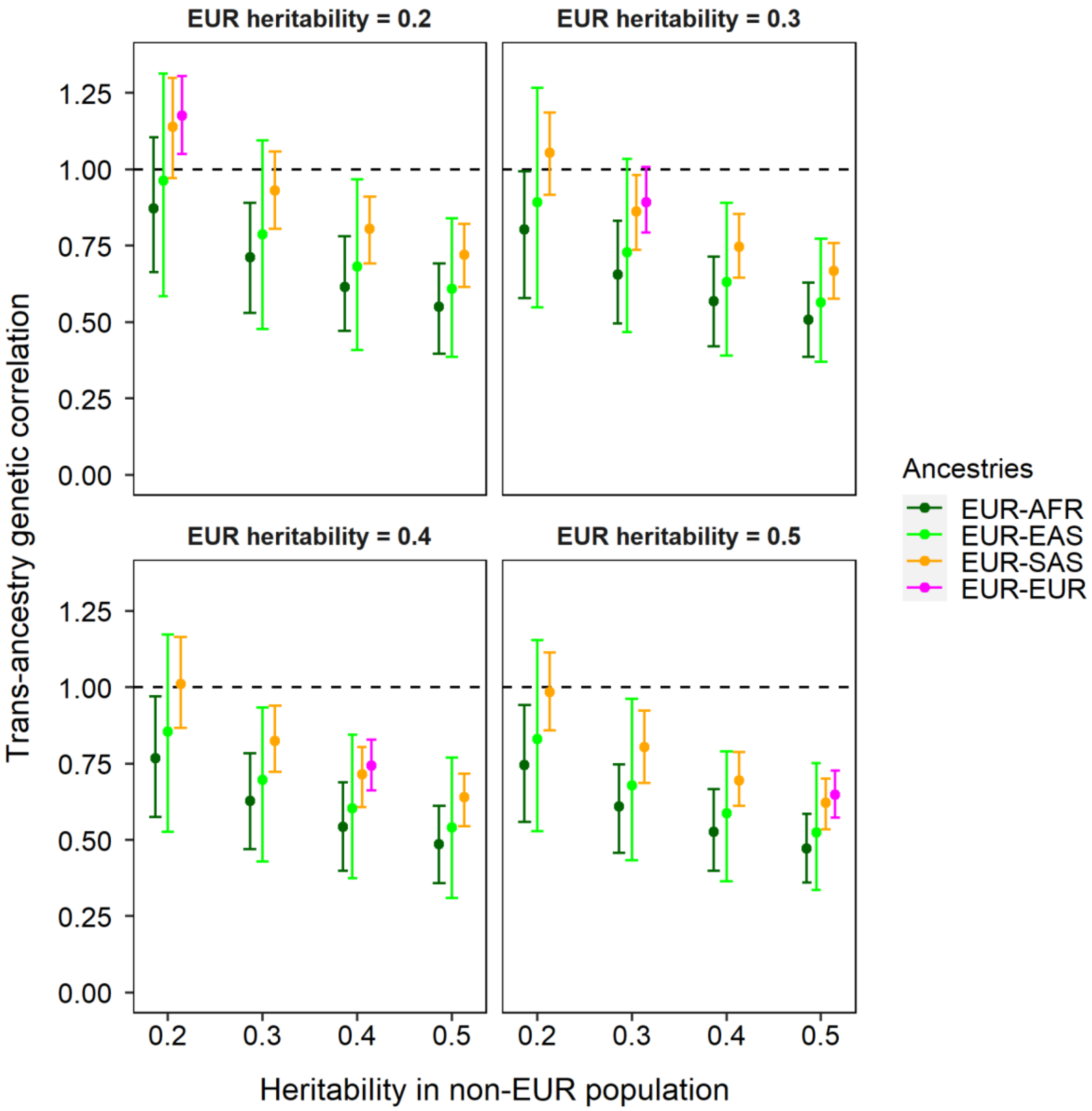

Supplement: Supplement 1 [file iovs-66-11-60_s001.pdf]
